# Nodular Scleritis in Association with Panuveitis in Behçet’s Disease 

**Published:** 2016

**Authors:** Ali Osman SAATCI, Ziya AYHAN, Fatos ONEN, Zeynep OZBEK, Ismet DURAK

**Affiliations:** 1 Professor, Dokuz Eylul University, Department of Ophthalmology, Izmir, Turkey; 2 Fellow, Dokuz Eylul University, Department of Ophthalmology, Izmir, Turkey; 3 Professor, Dokuz Eylul University, Department of Internal Medicine, Izmir, Turkey

**Keywords:** Behçet’s Disease, Scleritis, Uveitis

## Abstract

This case report involves a 32-year-old man with Behçet’s disease who had simultaneous bilateral anterior uveitis, unilateral nodular scleritis, and occlusive vasculitis with retinal hemorrhages. Although scleritis is not a classical feature of Behçet’s disease, a diagnosis of Behçet’s disease should be considered in patients with scleritis.

## INTRODUCTION

According to the Behçet’s Syndrome International Study Group Criteria, eye lesions in Behçet’s disease include anterior uveitis and posterior uveitis as well as cells in the vitreous on slit-lamp examination or retinal vasculitis observed by an ophthalmologist (1). Classically, Behçet’s uveitis presents as nongranulomatous panuveitis with occlusive periphlebitis and retinal hemorrhages (2). Unlike uveitis, scleritis is rarely reported among patients with Behçet’s disease (3-6). For example, in a group of 358 eyes of 266 patients with different types of scleritis, Behçet’s disease was the underlying systemic disorder in only one patient (7). We hereby report on a patient with bilateral panuveitis and simultaneous unilateral nodular scleritis.

## CASE REPORT

**Figure 1:**
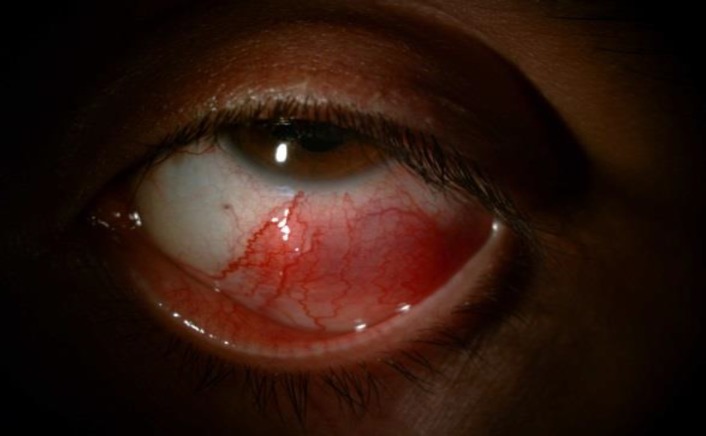
Appearance of nodular scleritis below the limbus of the left eye.

We examined a 32-year-old man who was diagnosed with bilateral anterior uveitis almost a year ago. His symptoms consisted of left eyeball pain and a decrease in vision in his left eye. He was treated with 1% topical prednisolone bilaterally three times a day by the referring ophthalmologist. He had experienced recurrent oral aphthous ulcers and papulopustular skin lesions for almost 10 years. He also had a history of lower extremity deep vein thrombosis. We found that the best-corrected visual acuity was 20/20 in the patient’s right eye and 20/40 in the left. A slit-lamp examination revealed trace cells in the anterior chamber and vitreous of the right eye, and in the left eye, tender scleral nodules with a purplish hue (Figure 1) and grade 2+ cells in the anterior chamber and vitreous.

**Figure 2:**
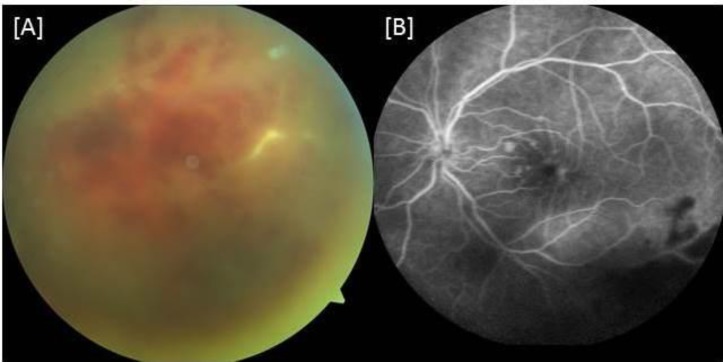
Lower inferior quadrant of the fundus of the left eye. (A) Occlusive vasculitis (B) Midphase fluorescein angiogram showing mild macular edema and masking of retinal architecture due to hemorrhagic vasculitis.

There was posterior synechia at 1 o’clock in the right eye and at 2 o’clock in the left eye. The intraocular pressure was within the normal limits bilaterally. While the right fundus was normal, there was grade 3 vitreous haze, according to the University of Miami Scale for Photographic Grading of Vitreous Haza (8), and occlusive vasculitis with intraretinal hemorrhages in the lower temporal quadrant of the left eye (Figure 2A). A fluorescein angiogram showed mild macular edema with findings related to occlusive vasculitis in the left eye (Figure 2B). The fluorescein angiogram of the right eye was normal. Optical coherence tomography indicated that both eyes had normal macular architecture.

**Figure 3:**
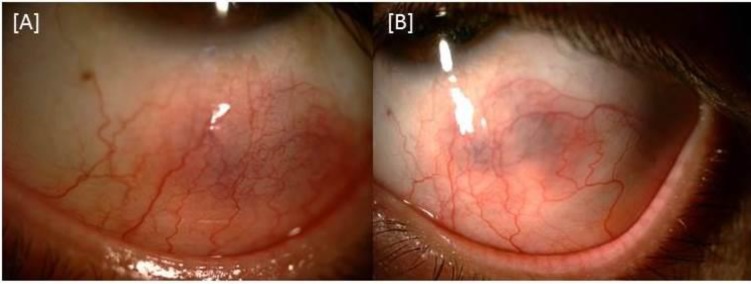
Appearance of the left eye (A) 1 week and (B) 3 weeks after the initiation of systemic treatment. The purplish hue corresponds to the area of nodular scleritis.

The patient underwent a detailed assessment by an experienced rheumatologist, which involved a systemic examination and a laboratory work-up. Varicose veins were observed on his lower extremities. The patient was diagnosed with Behçet’s disease associated with nodular scleritis and occlusive vasculitis in the left eye. Subsequently, the patient was treated with azathioprine (150 mg) and oral prednisolone (60 mg) along with 1% topical prednisolone bilaterally six times a day. The nodular scleritis resolved rapidly within a few weeks (Figures 3A and B) and, 3 weeks later, the patient’s visual acuity in the left eye was improved to 20/25, with a slight improvement in the appearance of fundus.

## DISCUSSION

Although characteristic ocular findings have been well-described for Behçet’s disease (9), scleritis has rarely been reported in patients with Behçet’s disease. Chang and Cho (3) described a 46-year-old woman with intestinal Behçet’s disease who developed bilateral nodular scleritis without any uveal involvement. The scleritis could only be controlled by using cyclophosphamide treatment. Dursun et al (4) reported on an 18-year-old girl with bilateral diffuse scleritis, lateral rectus myositis, and unilateral retrobulbar neuritis in association with neuro-Behçet disease. Again, the scleritis could only be controlled by using intravenous cyclophosphamide treatment. Sakellariou et al (5) described a 38-year-old woman with Behçet’s disease and unilateral scleromalacia perforans. Although the patient had bilateral anterior uveitis intermittently, no posterior segment involvement was noted. Remission could only be achieved with azathioprine and infliximab treatment. Very recently, Damodaran et al (6) reported on a 45-year-old woman with unilateral nodular scleritis. No posterior segment involvement or angiographic changes were present. The preferred treatment was a combination of prednisolone and azathioprine.

The unique feature of the patient with Behçet’s disease in this report is that he exhibited simultaneous nodular scleritis and occlusive vasculitis with extensive retinal hemorrhages. In light of this finding and those of the other recent reports, scleritis should also be considered, along with uveitis, among the ocular features of Behçet’s disease.
